# Caffeine Has Different Immunomodulatory Effect on the Cytokine Expression and NLRP3 Inflammasome Function in Various Human Macrophage Subpopulations

**DOI:** 10.3390/nu13072409

**Published:** 2021-07-14

**Authors:** Elek Gergő Kovács, Ahmad Alatshan, Marietta Margit Budai, Zsolt Czimmerer, Eduárd Bíró, Szilvia Benkő

**Affiliations:** 1 Departments of Physiology, Faculty of Medicine, University of Debrecen, H-4012 Debrecen, Hungary; kovacs.gergo@med.unideb.hu (E.G.K.); ahmad.alatshan@med.unideb.hu (A.A.); budaimarietta@gmail.com (M.M.B.); biro.eduard@med.unideb.hu (E.B.); 2 Doctoral School of Molecular Cellular and Immune Biology, Faculty of Medicine, University of Debrecen, H-4012 Debrecen, Hungary; 3 Departments of Immunology, Faculty of Medicine, University of Debrecen, H-4012 Debrecen, Hungary; 4 Department of Biochemistry and Molecular Biology, Faculty of Medicine, University of Debrecen, H-4032 Debrecen, Hungary; czimmerer.zsolt@med.unideb.hu

**Keywords:** caffeine, inflammation, macrophages, NLRP3 inflammasome, cytokines, signaling

## Abstract

Besides its well-known psychoactive effects, caffeine has a broad range of actions. It regulates several physiological mechanisms as well as modulates both native and adaptive immune responses by various ways. Although caffeine is assumed to be a negative regulator of inflammation, the effect on the secretion of pro- and anti-inflammatory cytokines is highly controversial. Macrophages are major mediators of inflammatory responses; however, the various subpopulations develop different effects ranging from the initiation to the resolution of inflammation. Here we report a comparative analysis of the effect of caffeine on two subpopulations of human monocyte-derived macrophages differentiated in the presence of macrophage colony-stimulating factor (M-CSF) or granulocyte-macrophage colony-stimulating factor (GM-CSF), resulting in M-MΦs and GM-MΦs, respectively. We showed that although TNF-α secretion was downregulated in both LPS-activated MΦ subtypes by caffeine, the secretion of IL-8, IL-6, and IL-1β as well as the expression of Nod-like receptors was enhanced in M-MΦs, while it did not change in GM-MΦs. We showed that caffeine (1) altered adenosine receptor expression, (2) changed Akt/AMPK/mTOR signaling pathways, and (3) inhibited STAT1/IL-10 signaling axis in M-MΦs. We hypothesized that these alterations play an important modulatory role in the upregulation of NLRP3 inflammasome-mediated IL-1β secretion in LPS-activated M-MΦs following caffeine treatment.

## 1. Introduction

Caffeine is a naturally occurring methylxanthine derivative that, as an antagonist of adenosine receptors, has impact on a wide range of physiological processes including the function of the nervous, cardiovascular, and immune systems. Besides being the most commonly consumed beverage, caffeine may be a component of various drug compounds [1,2], and it is also listed on the World Health Organization (WHO) Model List as a minimum medicine need for a basic health-care system [3]. As a therapeutic tool, caffeine is used to treat apnea of prematurity in preterm newborn infants, and it has been found to reduce bronchopulmonary dysplasia (BPD), a chronic lung disease of preterm infants, by reducing pulmonary inflammation, including IL-6 and TNF-α secretion [4,5]. In general, caffeine is assumed as an immunosuppressor agent as it inhibits proliferation, activation, and cytokine secretion by lymphocytes. Furthermore, caffeine is most often reported as a negative regulator of inflammatory responses due to its inhibitory effect on the secretion of TNF-α pro-inflammatory cytokine [6,7]. However, its effect on the secretion of other inflammatory cytokines, such as IL-6, IL-8, and IL-1β, is highly controversial, probably due to the different conditions, model systems, and cell types used in the studies [8].

One of the major sources of inflammatory cytokines are the macrophages that serve as a critical line of defense in innate immunity. However, depending on the origin and the local tissue microenvironment, macrophages develop to a broad spectrum of subpopulations that exhibit distinct phenotypic and functional features [9,10]. This way, the various macrophages serve inflammation at a wide range. While some subpopulations initiate and sustain inflammatory responses, other populations participate in the resolution of inflammation, help wound healing, and tissue regeneration [11].

Macrophages recognize pathogen-associated molecular patterns (PAMPs) and danger-associated molecular patterns (DAMPs) through pattern-recognition receptors (PRRs), and modify signal transduction pathways to regulate various cellular and immune mechanisms. The NOD-like receptor (NLR) family belongs to the cytosolic PRRs, and plays a critical role in innate immunity through the recognition of a broad range of molecular patterns. Beside signal pathway regulation, many of the NLR family members (such as NLRP1, NLRP3, NLRC4) function as sensors in multiprotein complexes called inflammasomes that are responsible for the maturation process of IL-1β conductor cytokine [12,13]. The most studied inflammasome complex is the NLRP3 inflammasome that requires two signals for its function. A priming signal, through TLRs or cytokine receptors, triggers the expression of the inflammasome components and that of the pro-IL-1β, while the activation signals that may emerge from various PAMPs or DAMPs lead to the assembly of the complex and to the activation of caspase-1 that subsequently cleaves IL-1β to its matured form [14,15,16]. Although the dysregulation of NLRP3 inflammasome activation is implicated in many acquired inflammatory diseases, it is required to promote host immune defense against pathogenic infections and danger signals [17,18]. Moreover, NLRP3 inflammasome-mediated IL-1β has been shown as the major driver of sterile inflammation that accompanies metabolic and autoinflammatory diseases as well as aging, and results in the development of low-grade, chronic inflammatory processes [19]. Due to the indispensable functions of NLRP3 inflammasome in inflammatory mechanisms, this multiprotein complex has become an important therapeutic target of various research [20].

Although NLRP3 inflammasome functions have been extensively studied, and the immunomodulatory effect of caffeine has been known for a while, the potential modulatory role of caffeine on the NLRP3 inflammasome-mediated IL-1β production on various macrophage populations has not been reported before. Here, we described for the first time the comparative analysis of the effect of caffeine on human monocyte-derived macrophages differentiated in the presence of either macrophage colony-stimulating factor (M-CSF) or granulocyte-macrophage colony-stimulating factor (GM-CSF), resulting in the development of inflammation resolving (M-MΦs) or inflammation promoting (GM-MΦs) macrophages, respectively. Our results show that caffeine modulates the different subpopulations of macrophages in different ways. While it downregulates TNF-α secretion in both, and it does not affect pro-inflammatory cytokine secretion in GM-MΦs, it significantly upregulates IL-6, IL-8, and IL-β production in M-MΦs. We showed that in both resting and activated M-MΦs, the expression of several members of the NLR family is induced by caffeine. We also provided mechanistic evidence for the caffeine-induced prolongation of NLRP3 inflammasome-mediated IL-1β secretion in M-MΦs. We showed that caffeine modulates adenosine receptor expression, and Akt/AMPK/mTOR signaling pathways. Furthermore, it inhibits the STAT1/IL-10 regulatory axis in M-MΦs. Altogether, our results indicate that caffeine drives cell specific immunomodulatory effects. Furthermore, these results also showed that caffeine modulates several factors that may intervene with NLRP3 inflammasome activation, resulting in an enhanced and prolonged IL-1β secretion by the LPS-activated M-MΦs.

## 2. Materials and Methods

### 2.1. Reagents

Ultrapure LPS from E. coli was purchased from InvivoGen (San Diego, CA, USA). Adenosine 5′-triphosphate, disodium salt (ATP) and caffeine were obtained from Sigma–Aldrich (St. Louis, MO, USA). Recombinant human IL10, M-CSF was obtained from PeproTech (Rocky Hill, NJ, USA), GM-CSF (Gentaur Molecular Products, Brussels, Belgium). MCC950 (NLRP3-inflammasome inhibitor) was obtained from InvivoGen (San Diego, CA, USA), and Z-YVAD-FMK (caspase-1 inhibitor) from BioVision Technologies (Milpitas, CA, USA).

### 2.2. Monocyte Isolation and Macrophage Differentiation

Leukocyte-enriched buffy coats were obtained from healthy blood donors in accordance with the written approval of the Director of the National Blood Transfusion Service and the Regional and Institutional Ethics Committee of the University of Debrecen, Medical and Health Science Center (Debrecen, Hungary). The informed consents of all participating subjects were obtained. Monocytes were isolated as previously described [21]. In brief, human peripheral blood mononuclear cells (PBMCs) were isolated by density-gradient centrifugation using Ficoll Paque PLUS (GE Healthcare Life Sciences, Little Chalfont, United Kingdom), followed by negative selection for CD14 positive cells using anti-CD14-conjugated microbeads (Miltenyi Biotec, Bergisch Gladbach, Germany). The obtained cells were cultured in RPMI 1640 (Sigma–Aldrich, St. Louis, MO, USA) containing 2 mM of L-glutamine and supplemented with 10% heat-inactivated-FBS and 500 U/mL of penicillin-streptomycin (Life Technologies, Carlsbad, CA, USA). For monocyte experiments, the cells were plated for 2 h to attach, then subjected to the treatments. For macrophages, the cells were differentiated for 5 days in the presence of either 50 ng/mL of M-CSF (Peprotech, London, UK) to obtain M-MΦs, or 80 ng/mL of GM-CSF (Gentaur Molecular Products, Brussels, Belgium) to obtain GM-MΦs, and on day 2, M-CSF or GM-CSF was replenished. Finally, the cells were treated with caffeine (300–1000 µM) then primed with LPS (100 ng/mL) for the indicated time points. For the inflammasome activation, the cells were subjected to media supplemented with ATP (5 mM) for 45 min.

### 2.3. Cytokine Measurements, Enzyme-Linked Immunosorbent Assay (ELISA)

The secretion level of the of the cytokines were measured from the cell culture supernatants. The collected supernatants were centrifuged and stored at −20 °C until further use. IL-6, TNF-α, IL10, and IL-8 were detected from ATP-free supernatants, while IL-1β was measured after ATP treatment, using ELISA kits (BD Biosciences, San Diego, CA, USA) in accordance with the manufacturer’s instructions. The measurements were performed by a FlexStation 3 Microplate Reader (Molecular Devices, Sunnyvale, CA, USA). The minimum detectable doses for the kits were as follows, 0.8 pg/mL for IL-1β, 2 pg/mL for IL-10 and TNF-α, and 2.2 pg/mL for IL-6 and IL-8.

### 2.4. RNA Preparation and RT-PCR

Total RNA was isolated from the cell pellets using TriReagent (UD-GenoMed, Debrecen, Hungary) in accordance with the manufacturer’s instructions. Spectrophotometer (NanoDrop ND1000; Promega Biosciences, Madison, WI, USA) was used to determine the concentration and homogeneity of total RNA. Calculated amounts of RNA were digested with DNase (Ambion, Austin, TX, USA) then subjected to reverse-transcription to obtain cDNA using SuperScript II first-strand reverse transcriptase and oligo dT primers (Thermo Fisher Scientific, Waltham, MA, USA).

### 2.5. Quantitative Real-Time Polymerase Chain Reaction (qPCR)

The measurement of gene expression was performed by subjected the cDNA to PCR reaction using a QuantStudio12K Flex qPCR instrument (Applied Biosystems). In the amplification reaction, TaqMan™ Gene Expression Master Mix and TaqMan Gene Expression Assays were used (Thermo Fisher Scientific, Waltham, MA, USA) (Table 1). For this reaction, the conditions were 40 cycles for 2-stage PCR (95 °C for 12 s and 60 °C for 1 min). The comparative Ct method was used to calculate the expression level for each transcript, and human cyclophilin (Ppia) was used for normalization as internal control.

### 2.6. Western Blot Analysis

The harvested cells were lysed and resuspended in loading buffer (62.5 mM Tris- HCl, pH 8.8, containing 25% glycerol, 2% SDS, 1% b-mercaptoethanol, and 1% bromophenol blue), then boiled at 100 °C for 10 min. The supernatant proteins were precipitated 20% TCA then boiled in loading buffer. The samples containing total proteins were subjected to SDS-PAGE and transferred onto nitrocellulose membranes. For blocking, the membranes were incubated in 5% nonfat milk in TBST and probed with primary antibodies at 4 °C overnight (Table 2). After washing, the membranes were incubated with horseradish peroxidase-conjugated secondary antibodies in blocking buffer for 1 h at room temperature. Membrane-bound peroxidase proteins were visualized by the ECL system (SuperSignalWest Pico/Femto chemiluminescent substrate; Thermo Fisher Scientific, Waltham, MA, USA). For loading control, β-actin protein expression was used.

### 2.7. Statistical Analysis

Experimental results are shown as the means ± SEM. (* *p* < 0.05, ** *p* < 0.01, *** *p* < 0.001). Statistical significance was determined by analysis of variance (ANOVA) with a Tukey–Kramer test, with the minimum donor number of 3.

## 3. Results

### 3.1. Caffeine Differently Modulates Cytokine Secretion by LPS-Activated Human Myeloid Cells

**Figure 1:**
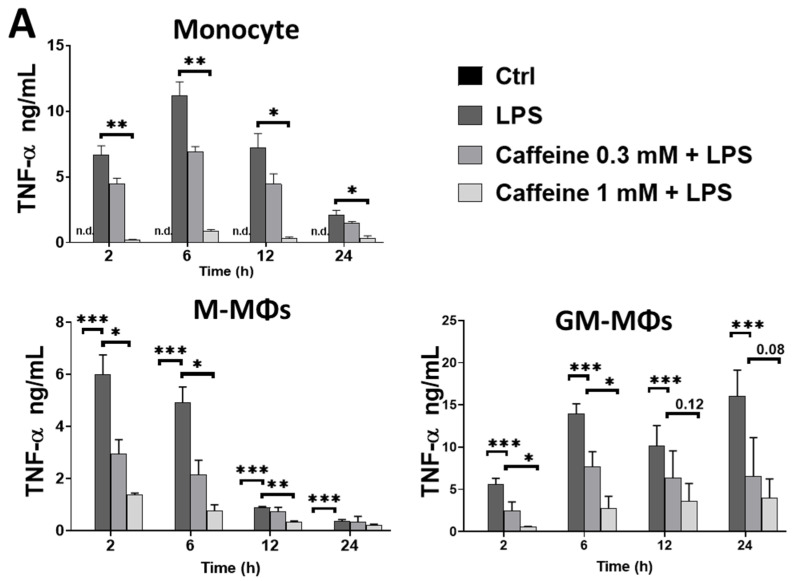

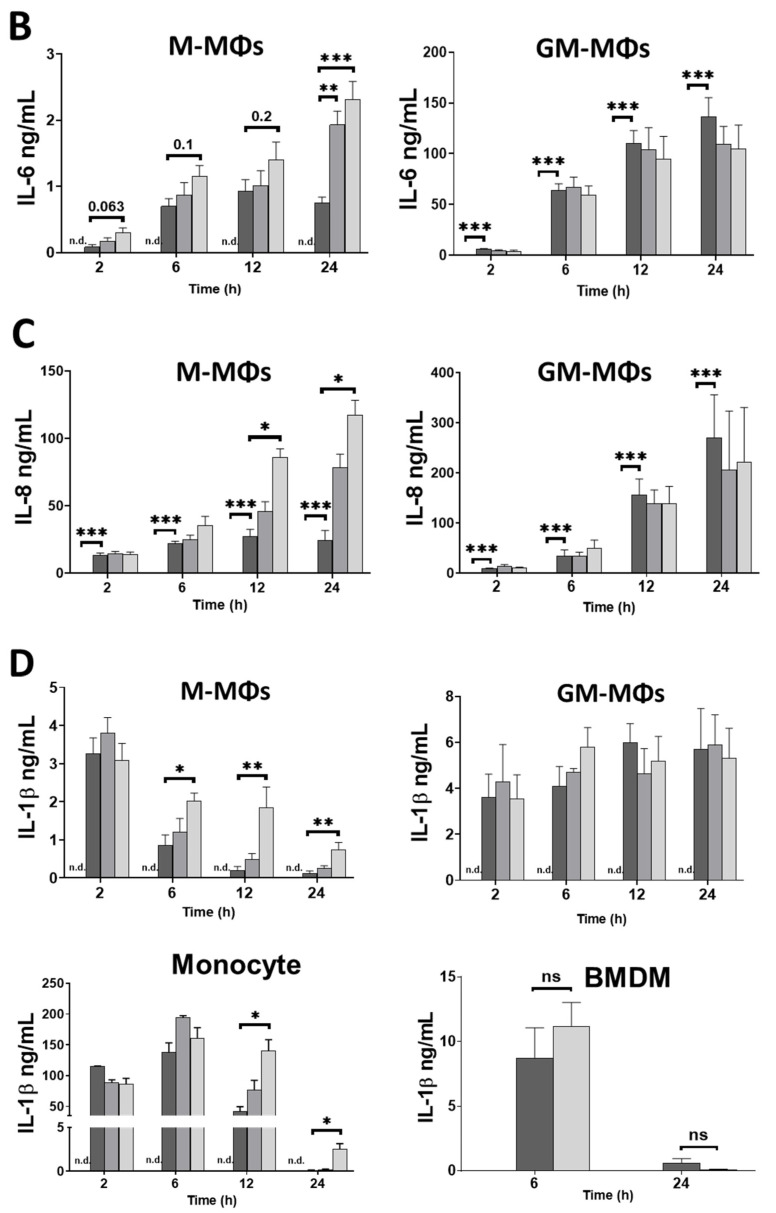
The modulatory effects of caffeine on cytokines secretion in different LPS-activated human myeloid cells. Cells were pre-treated with caffeine (0.3-1 mM) for 1 h, and then stimulated with LPS (100 ng/mL) for the indicated time. Cell culture supernatants were collected, and the secretion of (**A**) TNF-α, (**B**) IL-6, (**C**) IL-8, and (**D**) IL-1β were measured by ELISA. For IL-1β induction, cells were incubated with ATP (5 mM) for 45 min after LPS exposure. All results are shown as means ± SEM. (* *p* < 0.05, ** *p* < 0.01, *** *p* < 0.001).

To attain a better understanding of the effect of caffeine on the pro-inflammatory cytokine secretion, we activated different types of myeloid cells with LPS in the absence or presence of caffeine and measured secreted cytokines from the culturing medium using ELISA method. Caffeine treatment alone did not change any of the cytokine secretion by the cells (data not shown). We found that caffeine pre-treatment significantly decreased TNF-α secretion by monocytes and by both human macrophage subtypes (Figure 1A). Interestingly however, we found that while caffeine pre-treatment did not change the IL-6 (Figure 1B), IL-8 (Figure 1C), and IL-1β (Figure 1D) secretion by the LPS-activated GM-MΦs, the amount of these cytokines was significantly elevated in the medium of activated M-MΦs in the presence of caffeine compared to those cells that were activated in the absence of caffeine. Similarly, enhanced IL-1β secretion was measured from human, LPS-activated monocytes, while we did not observe significant changes in IL-1β secretion in the case of mouse bone marrow-differentiated MΦs (BMDMs) that were differentiated in the presence of M-CSF (Figure 1D). Of note, using the MTT assay, we did not detect changes in cell viability under the treatment conditions applied (data not shown). These results showed that caffeine has an immunomodulatory effect on myeloid cells; however, importantly, caffeine differently modifies LPS-induced cytokine secretion in the various populations of myeloid cells.

### 3.2. Caffeine Enhances the Expression of Many Intracellular Pattern-Recognition Receptors in M-MΦs but Not in GM-MΦs

**Figure 2:**
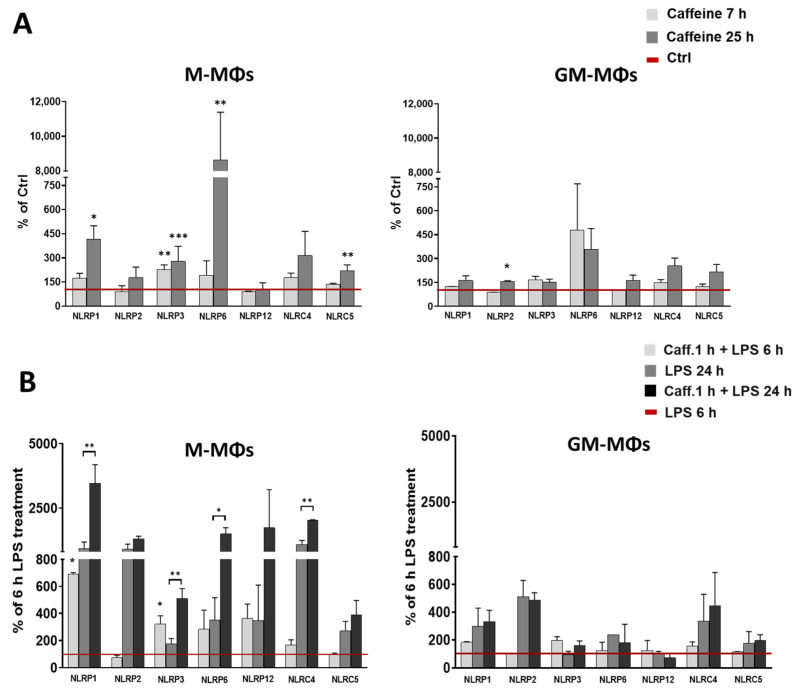
Effects of caffeine on NLRs expression in M-MΦs and GM-MΦs. The relative gene expression of indicated genes was measured by qPCR. (**A**) Cells were treated with caffeine (1 mM) for different time points. (**B**) Cells were pre-incubated with caffeine (1 mM) for 1 h, then primed with LPS (100 ng/mL) for the indicated time. Cyclophilin (Cyclo) was used as reference gene to normalize the gene expression. All results are shown as means ± SEM. (* *p* < 0.05, ** *p* < 0.01, *** *p* < 0.001).

Macrophage activation and inflammatory cytokine expression may be triggered and regulated by a wide spectrum of intracellular pattern-recognition receptors (PRRs), including Nod-like receptors (NLRs), either by modulating signal transduction pathways, or by forming inflammasome protein complexes to regulate IL-1β secretion. To see whether caffeine has effect on the expression of these sensor proteins, cells were treated with caffeine and changes in expression were measured using the qRT-PCR method. In the case of GM-MΦs, though the expression of NLRs showed a slight tendency of increase, none of them showed significant changes except NLRP2 (Figure 2A). Nevertheless, in the case of M-MΦs, we measured significant increase in the expression of NLRP1, NLRP3, NLRP6, and NLRC5 following a 25-h caffeine treatment. We obtained similar results in the case of LPS-activated cells; caffeine pre-treatment did not change significantly the expression of the NLRs in GM-MΦs, while the expression of NLRP1, NLRP3, NLRP6, and NLRC4 was significantly enhanced in the 24 h treated M-MΦs (Figure 2B). Of note, NLRP3 was the only NLR of which expression was highly affected by caffeine alone or in combination with LPS both at an early (6 h) and a late time (24 h) point. Our results altogether show that caffeine differently effects the expression of NLRs in the two macrophage subpopulations. While caffeine sensitizes M-MΦs by enhancing the expression of sensory molecules, it does not have significant effect on the NLR expressions in GM-MΦs.

### 3.3. Caffeine Pre-Treatment Enhances the Expression and Activation of NLRP3 Inflammasome in M-MΦs

**Figure 3:**
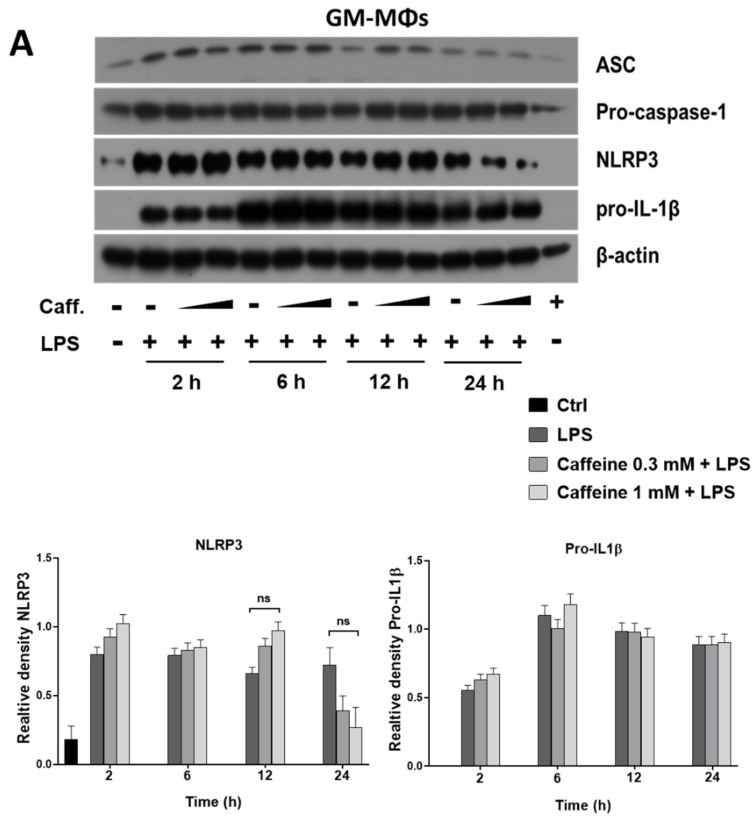

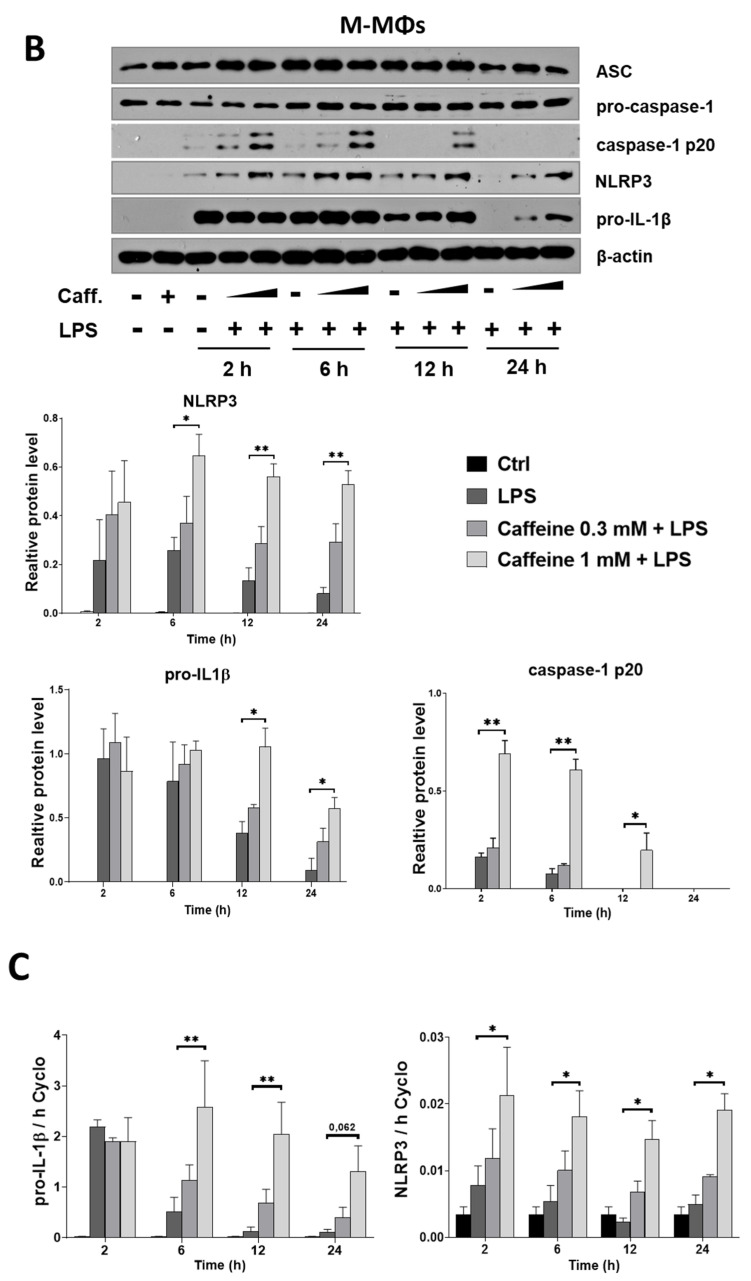

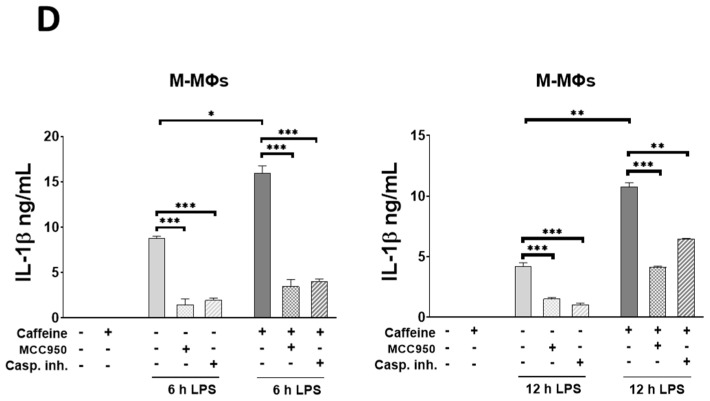
Effects of caffeine pre-treatment on the expression and activation of NLRP3 inflammasome in MΦs. Cells were pre-treated with caffeine (0.3–1 mM) for 1 h, then stimulated with LPS (100 ng/mL) for the indicated time. Representative immunoblots of ASC, pro-caspase-1, NLRP3, pro-IL-1β, and Caspas-1 p20 obtained from cell lysates of GM-MΦs (**A**) and M-MΦs (**B**). Bar graphs represent the relative protein expression of pro-IL-1β and NLRP3 and Caspas-1 p20 determined by densitometry (**B**). The relative gene expression of pro-IL-1β and NLRP3 of LPS-activated M-MΦs (**C**). M-MΦs were pre-treated with caspase-1 inhibitor (Z-YVAD-FMK, 20 mM) prior to LPS priming, or MCC950 (1 µM) 1 h before ATP treatment (**D**). Cyclophilin (Cyclo) was used as reference gene to normalize the gene expression. All results are shown as means ± SEM. (* *p* < 0.05, ** *p* < 0.01, *** *p* < 0.001).

NLRP3 inflammasome is a multiprotein complex that mediates IL-1β secretion in both sterile- and pathogen-related inflammatory responses [20]. As upon caffeine treatment, LPS-activated M-MΦs showed prolonged IL-1β secretion and significantly enhanced expression of NLRP3, we aimed to study whether the expression of the components of NLRP3 inflammasome is affected by caffeine pre-treatment. Using Western blot, we did not detect significant changes in the expression of NLRP3, ASC, pro-caspase-1, or pro-IL-1β proteins in GM-MΦs. (Figure 3A). In the case of M-MΦs, we found that the protein expression of the ASC adaptor and pro-caspase-1 was not affected by any of the treatments (Figure 3B). However, pre-treatment of caffeine resulted in a significant and concentration dependent elevation of NLRP3 and pro-IL-1β expression in the LPS-activated M-MΦs both at protein level (Figure 3B) and mRNA level (Figure 3C). Moreover, compared to the cells treated only with LPS, the presence of caffeine resulted in stronger bands of the cleaved (p20), active form of caspase-1 enzyme in the activated M-MΦs (Figure 3B), indicating that caffeine modifies not only the expression, but also the activation of NLRP3 inflammasome.

To further verify the role of NLRP3 inflammasome, we carried out the experiments on M-MΦs in the presence of the caspase-1 specific inhibitor, as well as in the presence of MCC950, a specific inhibitor of NLRP3. Our results show that both inhibitors significantly downregulated IL-1β secretion (Figure 3D).

These results show that caffeine modulates the expression of the NLRP3 inflammasome components. Moreover, it also enhances the activation of the complex in M-MΦs, while it has no effect on its expression and activation in the LPS-treated GM-MΦs.

### 3.4. Caffeine Downregulates Akt Signaling, and Differently Regulates mTOR and AMPK Signaling Pathways in the M-MΦs and GM-MΦs

**Figure 4:**
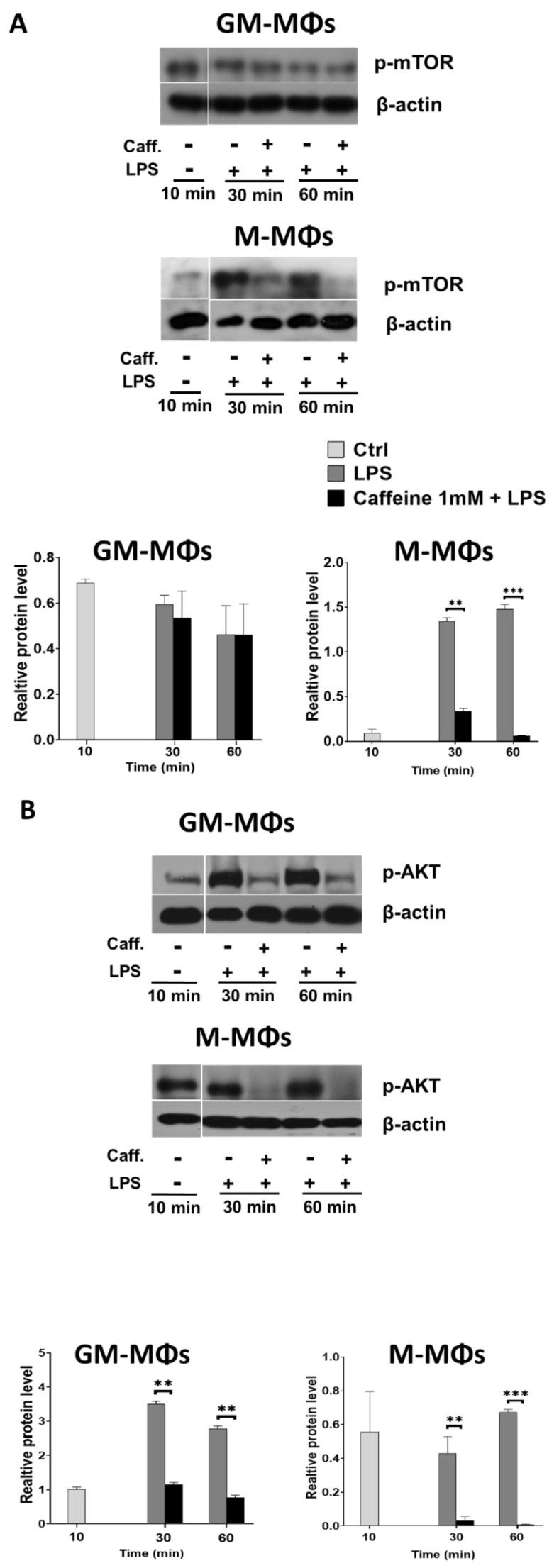

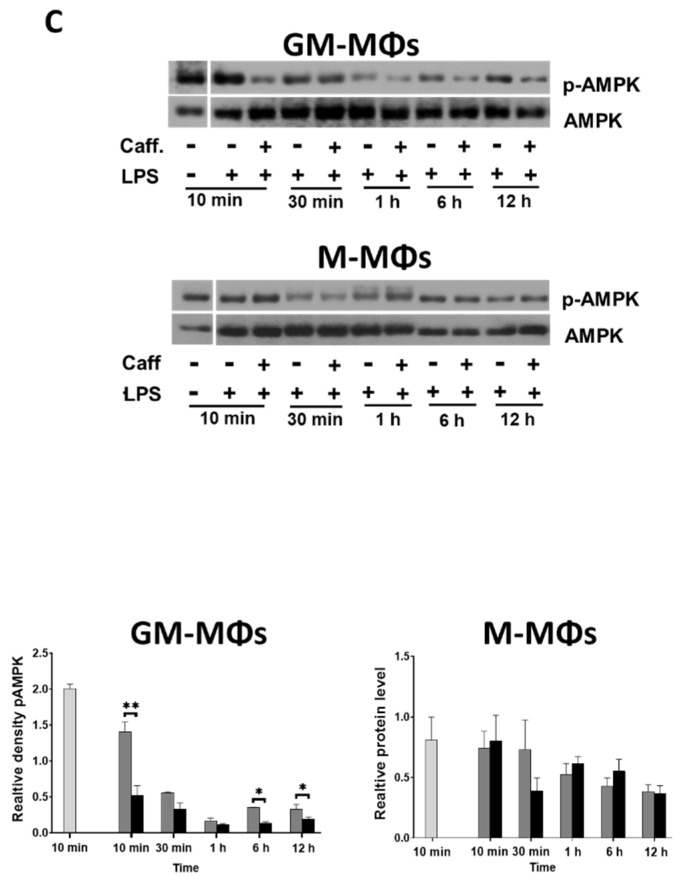
Caffeine differently regulates mTOR and AMPK signaling pathways in the M-MΦs and GM-MΦs. Cells were pre-treated with caffeine (1 mM) for 1 h, then stimulated with LPS (100 ng/mL) for the indicated time. Representative immunoblots of phosphorylated mTOR (**A**), AKT (**B**), and AMPK (**C**) obtained from cell lysates of M-MΦs and GM-MΦs. Bar graphs represent the relative protein expression determined by densitometry. β-actin was used as the internal control for normalization. All results are shown as means ± SEM. (* *p* < 0.05, ** *p* < 0.01, *** *p* < 0.001).

NLRP3 inflammasome activation may be regulated by a wide range of factors. To delineate possible mechanisms that caffeine exerts on NLRP3 inflammasome activation and IL-1β cytokine secretion in activated M-MΦs, we aimed to study relevant pathways. Caffeine has been described as a negative regulator of various signaling pathways, including mTOR [22]. mTOR is among the downstream targets of TLR4 signaling, and it was reported as a modulator of NLRP3 inflammasome activation by inhibiting caspase-1 activity [23]. Using cell lysates of LPS-activated MΦs, we found that caffeine had no significant effect on mTOR phosphorylation in GM-MΦs; while under the same condition, caffeine significantly downregulated the LPS-induced mTOR phosphorylation in M-MΦs (Figure 4A).

mTOR phosphorylation may be regulated via various upstream regulatory signals. As Akt was reported to enhance, while AMPK was reported to inhibit mTOR activation [24]. Using the Western blot method, we found that the Akt phosphorylation was abolished both in GM-MΦs and M-MΦs by caffeine (Figure 4B). However, we found differences in AMPK phosphorylation between the two macrophage subpopulations. Interestingly, while we observed a tendency to inhibit LPS-induced phosphorylation of AMPK in the presence of caffeine in GM-MΦs, in the M-MΦ subpopulation AMPK phosphorylation was not affected (Figure 4C).

These results together suggest that the different AMPK signaling together with the reduced Akt signaling may alter mTOR phosphorylation. Abolished mTOR phosphorylation may release its inhibitor effect on NLRP3 inflammasome in M-MΦs, which, in part, explains the different IL-1β secretion by the two MΦ subpopulations.

### 3.5. Caffeine Modifies Adenosine Receptor Expression

**Figure 5:**
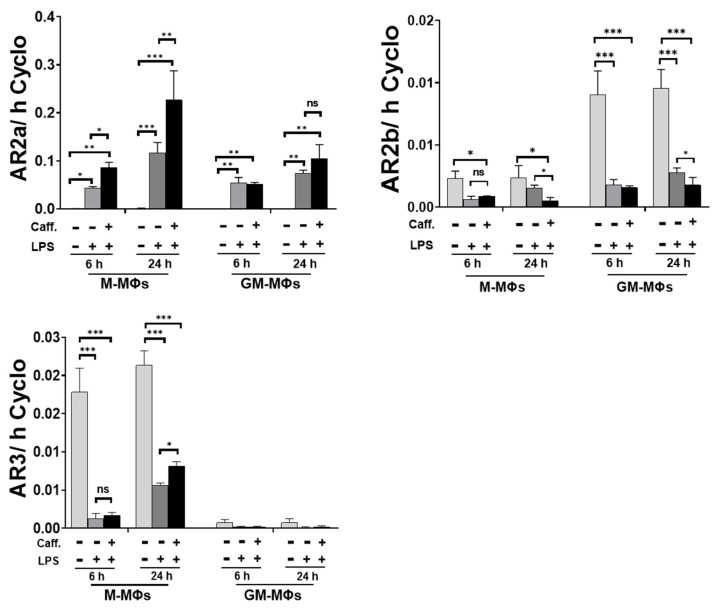
Caffeine modulates adenosine receptors expression. Cells were pre-treated with caffeine (1 mM) for 1 h, then stimulated with LPS (100 ng/mL) for the indicated time. The relative gene expression of adenosine receptors (AR2a, AR2b, and AR3). Cyclophilin (Cyclo) was used as reference gene to normalize the gene expression. All results are shown as means ± SEM. (* *p* < 0.05, ** *p* < 0.01, *** *p* < 0.001).

Caffeine functions as a non-selective antagonist of adenosine receptors (AR). ARs may regulate adenylate cyclase (AC) activity, thus changing ATP/AMP ratio in the cell, and eventually leading to the activation of AMPK signaling [25]. Hence, we aimed to measure the effect of caffeine on the expression of ARs on LPS-activated cells. We found that while caffeine significantly enhanced the expression of AR2a expression in activated M-MΦs), it did not have significant effect on the LPS-activated GM-MΦs (Figure 5). Furthermore, although we detected a higher basal expression of AR2b in GM-MΦs compared to M-MΦs, LPS activation significantly reduced the expressions in both cases. Pre-treatment with caffeine further decreased AR2b expressions in both activated MΦ subpopulations but only at the later time points (Figure 5). Interestingly, under resting condition, AR3 expression is more pronounced in M-MΦs compared to GM-MΦs where it is hardly detectable. Nevertheless, LPS treatment robustly downregulated AR3 expression in M-MΦs, but the presence of caffeine slightly relieved this inhibition at the later time point (24 h) (Figure 5). These results show that caffeine significantly modifies the expression of ARs, especially that of the AR2a, possibly highly effecting the ATP/AMP ratio in the activated MΦs subpopulation.

### 3.6. Caffeine Potentiates NLRP3 Inflammasome-Mediated IL-1β Secretion by Downregulating STAT1/IL-10 Axis in M-MΦs

**Figure 6:**
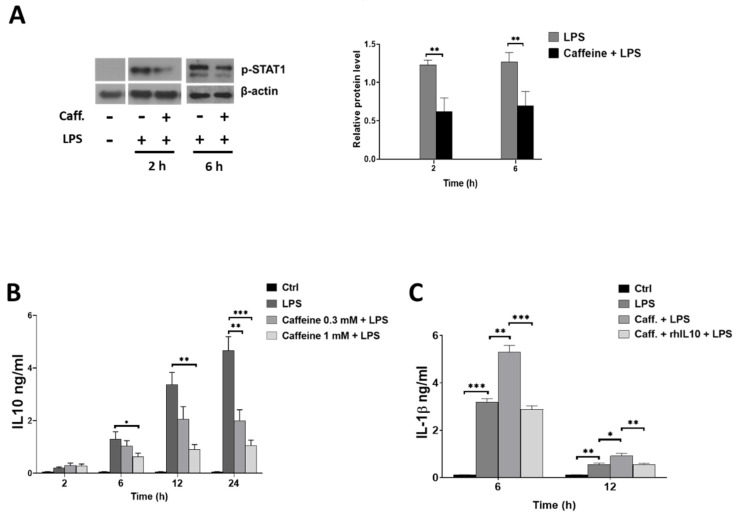
Caffeine modulates IL-10 and STAT1 signaling in M-MΦs. Cells were pre-treated with caffeine (0.3 mM or 1 mM) for 1 h, then stimulated with LPS (100 ng/mL) for the indicated time. (**A**) Representative immunoblots of phosphorylated STAT1 obtained from cell lysates of M-MΦs and bar graph represents the relative protein expression determined by densitometry. (**B**) IL-10 secretion was determined by ELISA from cell culture supernatants. (**C**) The cells were pre-treated with recombinant human IL-10 (rhIL-10) (100 ng/mL) 1 h before LPS activation and IL-1β secretion was determined by ELISA from cell culture supernatants. All results are shown as means ± SEM. (* *p* < 0.05, ** *p* < 0.01, *** *p* < 0.001).

Beside the classical pro-inflammatory cytokines, in M-MΦs, LPS induces the secretion of IL-10 anti-inflammatory cytokines, which, at the same time, act as negative regulator of NLRP3 inflammasome activation [26]. We have shown previously that IL-10 secretion is under the detection limit in GM-MFs (Budai et al., JLB, 2016); thus, we aimed to see the effect of caffeine on the expression of IL-10 and its upstream regulator STAT1 signaling pathway in LPS-activated M-MΦs, using ELISA and Western blot methods, respectively. We found that in the presence of caffeine, the LPS-induced STAT1 phosphorylation was significantly downregulated (Figure 6A). Furthermore, caffeine significantly reduced the IL-10 secretion in the LPS-activated M-MΦs (Figure 6B). To prove the effect of IL-10 on IL-1β secretion, we pre-treated the caffeine plus LPS-treated cells with human recombinant IL-10 cytokine. We found that adding recombinant IL-10 (rhIL-10) significantly alleviated the activation and reduced IL-1β secretion (Figure 6C). These results altogether suggest that the inhibitory effect of STAT1/IL-10 axis on NLRP3 inflammasome activation is attenuated by caffeine, and these changes participate in the enhanced secretion of IL-1β by M-MΦs.

## 4. Discussion

Caffeine (1,3,7-trimethylxanthine) is a member of the methylxanthine family of drugs, and probably one of the most frequently consumed compounds worldwide. Over the years, caffeine has been extensively studied, and a number of reports have shown that besides its psychoactive effects, caffeine also has impacts on various biochemical and physiological processes including the cardiovascular, respiration, or digestive systems [27]. Furthermore, a number of in vitro and in vivo studies have also proven that caffeine has an important immunomodulatory role effecting both the innate and the adaptive immune responses [8,28]. It has been shown that caffeine inhibits lymphocyte proliferation, antibody production, NK cell function, and chemotaxis [28,29,30,31], as well as histamine release, free radical production, and cytokine secretion by various cells [32,33]. Based on these reports, caffeine has been suggested as a negative regulator of immune responses [7,34], and mediates anti-inflammatory effects [35,36].

In our study, regarding TNF-α secretion, we observed an inhibitory effect of caffeine on the LPS-activated human monocytes, as well as on two, monocyte-derived macrophage subpopulations differentiated in the presence of M-CSF or GM-CSF. This observation is in good line with previous studies where caffeine was reported as a negative regulator of TNF-α secretion in various cells including splenocytes, whole blood [6], mast cells, or monocytes [31,37]. Similar results have been reported regarding MΦs, as it was shown that caffeine decreased TNF-α secretion from Kupffer cells isolated from ethanol-fed mice [38], and in microglia following retinal ischemia-reperfusion [39].

However, reports are highly contradictory regarding the effect of caffeine on other pro-inflammatory cytokines. As in whole blood, it has been shown that caffeine does not change the LPS-induced secretion of IL-10, IL-6, and IL-1β [6], while reduced mRNA expression of IL-6, IL-12, and IL-3 has been found in LPS-activated RAW264.7 cells [40] or retinal ischemia/reperfusion-induced IL-6 and IL-1β secretion by microglia [39,41]. Myeloid cells, especially macrophages, are essential mediators of inflammatory responses. However, depending on the local microenvironment and the activating stimuli, macrophages develop and polarize to different subpopulations [42,43]. This way they provide a repertoire of macrophages with a wide spectrum of effector functions, including cytokine secretion ranging from pro-inflammatory to anti-inflammatory ones. Macrophages differentiated in the presence of GM-CSF (GM-MΦs) are considered as inflammatory-type macrophages that help to initiate and maintain inflammation by producing high level of pro-inflammatory cytokines. Nevertheless, macrophages differentiated by M-CSF (M-MΦs) have important role in the termination of inflammation, and help wound healing and tissue regeneration in part by producing IL-10 anti-inflammatory cytokine [44]. Here we show that the effect of caffeine on the expression of cytokines by myeloid cells is cell type dependent. We found that, with the exception of TNF-α, caffeine had no modulatory effect on the secretion of pro-inflammatory cytokines by LPS-activated GM-MΦs. Importantly, however, it significantly elevated or prolonged the LPS-induced IL-6, IL-8, and IL-1β secretion by M-MΦs.

As the first line of defense in innate immunity, macrophages initiate and modulate cytokine secretion through various pattern-recognition receptors that sense and detect harmful pathogens or danger molecules. Currently, little is known about the effect of caffeine on the expression of PRRs. It was shown that in neonatal rat lung, postnatal caffeine treatment did not affect the expression of TLR2 and TLR4, but it significantly elevated the expression of TLR9 [45]. So far, there is only a single study regarding NLRs, and it showed that caffeine downregulated IL-1β secretion in adenine-treated THP-1 cells via the inhibition of NLRC4 [46]. NLRs interact with proteins of signal transduction pathways or form multiprotein complexes, called inflammasome, to modify cytokine secretion during immune responses [47]. Our results showed that caffeine had no effect on the NLR expression in GM-MΦs, while in M-MΦs it enhanced the expression of many of the inflammasome forming NLRs. Thus, by modulating NLR expression, caffeine may sensitize cells for danger/pathogen recognition in M-MΦs. Furthermore, elevated NLR expression may mediate a more pronounced cytokine response, as it is shown in the case of M-MΦs. Besides the inflammasome-forming NLRPs, our study shows that NLRC5 expression is also enhanced by caffeine. NLRC5 was reported to translocate to the nucleus [48] and induce MHC I expression by modulating the activity of transcription factors [49]. As caffeine has been reported to induce MHC I expression in T cells [50], our results give rise to the possibility that the caffeine-mediated upregulation of NLRC5 expression would mediate enhanced MHC I expression in M-MΦs. Nevertheless, this suggestion would require further detailed investigations.

**Figure 7:**
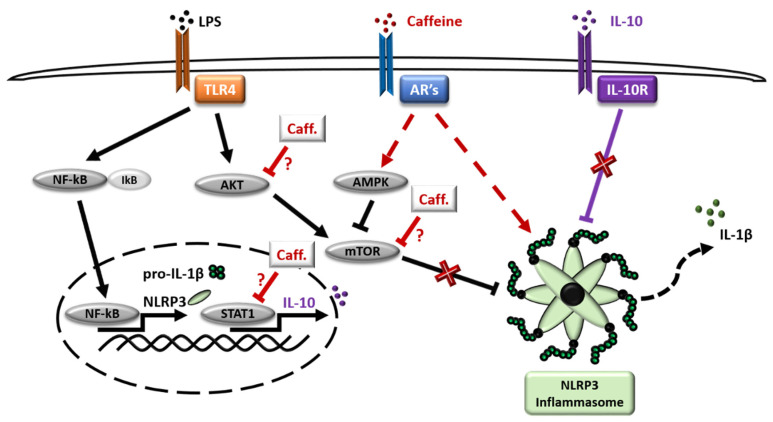
Schematic representation of caffeine effect on M-MΦs subpopulation. Cells were pre-treated with caffeine (1 h) and then stimulated with LPS. Red color represents caffeine effects, dashed line represents indirect effects.

We have previously described the different dynamics of pro-inflammatory cytokine secretion and the molecular mechanisms of NLRP3 inflammasome-mediated IL-1β secretion by the GM-MΦs and the M-MΦs [51]. In the present study, we showed that caffeine acts differently on NLRP3 inflammasome expression and activation in the two macrophage subpopulations. While it had no effect on the NLRP3-mediated IL-1β secretion in LPS-activated GM-MΦs, it enhanced and prolonged the expression of NLRP3 and pro-IL-1β, as well as the activity of caspase-1, consequently enhancing LPS-induced IL-1β secretion in the M-MΦs (Figure 7). Our results are in an apparent contradiction with a recent report, where caffeine treatment of LPS-activated THP-1 cell line resulted in the inhibition of NLRP3 inflammasome expression and reduced secretion of IL-1β due to the suppression of AR2a-associated ROS production [52]. Differences in our studies may be explained by the different model systems used, such as THP-1 versus blood monocyte-derived MΦs, as well as the time intervals of sample collections following LPS activation. Although THP-1 is widely used to model common NLRP3 inflammasome-related functions, the variable cellular and molecular mechanisms that characterize a specific MΦ subpopulation provide different factors to the complexity of NLRP3 inflammasome activation, which may explain the diverse, sometimes contradictory results.

Due to structural similarities, caffeine functions as a non-selective antagonist of adenosine receptors (ARs) (reviewed in [53]). Out of the 4 adenosine receptor subtypes, caffeine has the highest affinity to AR2a, which is the most important subtype in macrophages to mediate the immunomodulatory actions of adenosine [54]. Hence, its anti-inflammatory role is assumed to be vital in protecting the body against life-threatening inflammatory stimuli [55]. Our results showed different basal expressions of ARs between the two MΦ subpopulations. Furthermore, we found the most significant expression change caused by caffeine in the case of AR2a, as it was highly elevated in the activated M-MΦs compared to the GM-MΦs in the presence of caffeine both at early and later time points. Previously, the upregulation of ARs expression following caffeine treatment was reported in *Drosophila*; however, only one type of AR is expressed in flies, and that is homologous to vertebrate AR2b [56]. Furthermore, elevated expression of AR1 was shown in rats following caffeine treatment [57]. Nevertheless, based on our previous observation, AR1 is not expressed in M- or GM- MΦ [51].

The overall outcome of ARs expression change may be highly complex. In general, ligation of AR2a and AR2b with adenosine leads to the activation of adenylate cyclase (AC) enzyme, while activation of AR1 and AR3 has the opposite effect by inhibiting AC activity. Activation of AC converts ATP to cAMP, and eventually cAMP is converted to AMP by one of the PDE enzyme isoforms [58,59]. Changes in ATP/AMP ratio is an important modulator of AMPK signaling [60], while elevation of cAMP concentration was reported to suppress inflammatory responses [61], including inhibition of NLRP3 inflammasome activation [62]. Furthermore, caffeine may exert its effect not only through ARs, but it has also been reported as a direct inhibitor of PDE, and an activator of AC [63] (Figure 7). Moreover, the outcome of caffeine treatment and the activity of ARs is also affected by the available extracellular adenosine molecules, which is influenced by the expression and activity of ectonucleotidases (such as CD39 and CD73) [64] that converts extracellular ATP/ADP/AMP to adenosine, and even the presence and activity of adenosine transporters [64]. To find out the precise mechanism and participation of ARs to the observed effect of caffeine would require further detailed studies.

Interestingly, we detected obvious differences in mTOR phosphorylation between the two macrophage subpopulations. mTOR is an important mediator of several pathways including its regulatory/inhibitory effect on NLRP3 inflammasome activation [65]. mTOR phosphorylation and activation may be regulated via various upstream factors, including AMPK which inhibits mTOR, or Akt signaling which activates mTOR pathway [24]. Importantly, inhibition of Akt/mTOR signaling following caffeine treatment was reported in various cell types such as in osteosarcoma cells, hematopoietic myeloid cells, and SH-SY5Y cells [22,66,67]. Our results show that in the LPS-activated GM-MΦs, mTOR phosphorylation was not affected by caffeine pre-treatment, while reduced mTOR phosphorylation was detected in the presence of caffeine on LPS-activated M-MΦs. As Akt signaling was abolished in both LPS-activated MΦ subpopulations, we hypothesize that in GM-MΦs, due to the abolished inhibitory AMPK signaling, the LPS-induced mTOR pathway is not affected by caffeine, and the LPS-induced inflammasome activation is not changed (Figure 7). Furthermore, importantly, GM-CSF has been reported as an important stimulator of mTOR signaling; hence, this effect may also contribute to the observation that mTOR phosphorylation is not altered by caffeine in the GM-MΦs. Nevertheless, in M-MΦs, mTOR activity is inhibited by the active AMPK pathway or by caffeine itself, and released the mTOR inhibitory effect on NLRP3 inflammasome activation, in part, explaining the elevated IL-1β secretion in M-MΦs.

As a cytokine crosstalk mechanism, IL-1β production is modified by various other cytokines, such as how the anti-inflammatory IL-10 has been reported to develop inhibitory effect on the activation of NLRP3 inflammasome [68,69,70,71]. The pro-inflammatory-type GM-MΦs do not release detectable amounts of IL-10 following LPS activation [51]; however, in M-MΦs, we found that caffeine treatment inhibited the LPS-induced IL-10 secretion, as well as downregulating STAT1 signaling. STAT1 signaling is an important regulator of IL-10 cytokine production [72,73], and inhibition of mTOR signaling in macrophages was shown to inhibit IL-10 mRNA and protein expression [74,75]. Furthermore, caffeine has been reported to develop direct inhibitory effects on STAT1 signaling [41]. Hence, based on our results it seems that caffeine-enhanced IL-1β secretion in M-MΦs is, in part, mediated by the release of the inhibitory effect of STAT1/IL-10 axis (Figure 7).

## 5. Conclusions

Based on our results it seems that caffeine has a complex immunomodulatory effect on the human M-MΦs, including NLRP3 inflammasome-mediated IL-1β secretion. Differentiation and activation of macrophages result in a huge repertoire of functionally different subpopulations, both in human and mice. Furthermore, the activation of NLRP3 inflammasome is modulated at various levels by a wide spectrum of pathways and molecules. The spectrum of research that focuses on the effect of caffeine on the immune system has been exponentially growing during the last decade, and so has the confusion inferred from its contradictory results. Thus, it is possible that caffeine can elicit either anti-inflammatory or pro-inflammatory effects, depending on the dose employed, the concentration of endogenous adenosine present at the inflammatory site, and the model system used for the studies. Hence, to understand the cell type-specific effect of caffeine, and its potential therapeutic usage in inflammatory conditions and on NLRP3 inflammasome function in macrophages, detailed comparative analysis of the various MΦ subpopulations is required.

## Figures and Tables

**Table 1:** List of the gene expression assays.

Gene	Assay ID
Ppia (cyclophilin)	IDT (33731568) (33731567) (33731569)
IL-1β	Hs00174097_m1
NLRP1	Hs00248187_m1
NLRP2	Hs01546932_m1
NLRP3	Hs00918082_m1
NLRP6	Hs00373246_m1
NLRP12	Hs00536435_m1
NLRC4	Hs00368369_m1
NLRC5	Hs00260008_m1
AR2a	Hs00169123_m1
AR2b	Hs00386497_m1
AR3	Hs00181232_m1

**Table 2:** List of the used antibodies.

Antibody	CAT Number
Pro-IL-1β	12,703, Cell Signaling
IL-1β	83,186, Cell Signaling
Pro-caspase-1	3866, Cell Signaling
Caspase-1	4199, Cell Signaling
NLRP3	15,101, Cell Signaling
ASC	sc-30153, Santa Cruz Biotechnology
p-Akt/(S473)	9271, Cell Signaling
p-mTOR (Ser2448)	2971, Cell Signaling
p-STAT1 (Tyr701)	9167, Cell Signaling
p-AMPK	2535, Cell Signaling

## Data Availability

The data presented in this study are available on request from the corresponding author.
